# A Biomass Porous Carbon with Robust Salt Resistance Capacity for Continuously Efficient Solar-Driven Interfacial Desalination

**DOI:** 10.3390/ma19061172

**Published:** 2026-03-17

**Authors:** Pingping Liang, Xiaokun Wen, Shuai Liu, Wencui Xiu

**Affiliations:** 1 School of Mechanical and Civil Engineering, Jilin Agricultural Science and Technology College, Jilin 132101, China; xiuwencui@jlnku.edu.cn; 2 School of Mechanical and Electronic Engineering, Qingdao Binhai University, Qingdao 266555, China; wenxk782@nenu.edu.cn; 3 College of Science, Northeast Electric Power University, 169 Changchun Street, Jilin 132012, China; liushuai@neepu.edu.cn

**Keywords:** self-floating, superhydrophilicity, biomass-derived carbon, solar desalination, interfacial evaporation, salt rejection

## Abstract

Solar-driven vapor generation (SDVG) emerges as a promising solution to the global freshwater crisis; yet, the scalable applications in seawater desalination are significantly hindered by the salt deposition. Herein, we report a self-floating biomass porous carbon with robust salt resistance derived from a simple, one-step carbonization of the Elymus nutans. The material features a natural hierarchical pore structure and superhydrophilicity, which work synergistically to ensure a rapid water supply and effectively prevent salt crystallization at the evaporation interface. Under 1 sun illumination (1 kW m^−2^), the biomass-derived carbon achieves a high evaporation rate of 1.41 kg m^−2^ h^−1^ with a solar-to-vapor conversion efficiency of 88.9%. More importantly, it demonstrates exceptional stability, maintaining stable evaporation in 3.5 wt% and 15 wt% NaCl solutions for over 12 h, with recorded rates of 1.33 and 1.16 kg m^−2^ h^−1^, respectively. The real seawater sample desalinating results verified the biomass-porous-carbon-realized high-efficient and robust solar-driven interfacial desalination. This work presents a sustainable, cost-effective, and salt-resistant material platform, offering a practical pathway for scalable solar desalination.

## 1. Introduction

Fresh water is a core element for maintaining the balance of ecosystems and the development of human society. Nowadays, over 3 billion people worldwide are severely troubled by the shortage of fresh water, posing serious challenges to ecological security, social stability, and sustainable economic development [1,2,3]. Solar-driven vapor generation (SDVG), as an interfacial water treatment technology, has been recognized as a potential solution to the fresh water shortage. The water is delivered to the surface of the photothermal materials, and evaporates at the air/water interface by absorbing the heat from the materials [4,5,6]. It is well in accordance with the dual-carbon concept and brings new hope to those remote areas with power shortages and a weak infrastructure [7,8].

Seawater covers 71% of the total area of the earth. Seawater desalination has become the most economical technology to alleviate the freshwater crisis [9,10,11]. However, the high-concentration brine is more prone to form salt precipitates during the evaporation process than low-concentration wastewater. These salts always stack on the surfaces or the interfaces of the photothermal materials, shielding the light absorption and blocking the water transport channels. Such issues can significantly reduce the evaporation efficiency and even make the evaporators completely lose SDVG capability [12,13,14]. Therefore, developing photothermal materials with an excellent salt resistance capacity is urgently called for.

Constructing water transport channels within the materials to improve the water supply to the interface is a promising way to meet the requirement. Constructing porous channels, enhancing the hydrophilicity of materials, and improving the efficiency of solar energy absorption are the key to achieving salt deposition resistance and efficient and stable evaporation. Inspired by the water transport in the plants, the biomass materials have natural distinctive multi-level channels [14,15,16,17]. The capillary effect caused by this gradient structure enables water to be rapidly transported to the interfaces. Thus, the natural plant-derived biomass materials are quite suitable for SDVG. 

In this work, a self-floating and salt-resistant biomass material for SDVG was fabricated by the carbonization of Elymus nutans. The obtained biomass porous carbon retains its natural three-dimensional hierarchical porosity, exhibits excellent broadband light absorption, and demonstrates superhydrophilicity—collectively enabling continuous and efficient solar desalination. Owing to its interconnected pore network, the material achieves stable evaporation even in high-salinity brine (15 wt% NaCl) and real seawater over extended periods, without significant salt accumulation. This study not only presents a novel biomass-derived evaporator with exceptional salt rejection performance but also offers a practical and sustainable pathway toward scalable solar-driven freshwater production.

## 2. Materials and Methods

### 2.1. Materials

The Elymus nutans selected for the experiment was from Guinan County, Hainan Tibetan Autonomous Prefecture, Qinghai Province, China. Ethanol is purchased from Beijing Chemical Works (Beijing, China). Ultrapure water used in all experiments was prepared by the experimental ultrapure water system (LAB-IV-20) manufactured by Changchun Laibopate Technology Development Co., Ltd. (Changchun, China).

### 2.2. Material Preparation

The collected sun-dried stems of Elymus nutans were first washed with deionized water and ethanol, then dried at 60 °C for 24 h. The dried stems were placed in a tubular furnace and subjected to a controlled pyrolysis process under a continuous nitrogen flow (200 sccm). The temperature was ramped from room temperature to 400 °C at a rate of 5 °C min^−1^, held for 1 h for pre-carbonization and stabilization, and then further increased to 800 °C at the same ramp rate and maintained for 2 h to achieve complete carbonization into a carbon monolith. Finally, the sample was allowed to cool down naturally to room temperature under the N_2_ atmosphere. They are alternately washed three times with ultrapure water and anhydrous ethanol in sequence to thoroughly remove the residual impurities. The cleaned samples were placed in a vacuum-drying oven and dried at 60 °C for 12 h. The resulting biomass-derived carbon was denoted as BC; the Elymus nutans stems were denoted as ENS.

### 2.3. Characterization

The morphology of the BC was characterized by the scanning electron microscope (SEM, FEG-250, FEI Company, Hillsboro, OR, USA); the BC samples were directly placed on a conductive carbon tape and then subjected to a 60 s sputtering treatment with gold to enhance conductivity. The dynamic contact angle of the BC was measured using a contact angle goniometer (KRÜSS DSA100, Hamburg, Germany) at room temperature. A 1 μL water droplet was deposited directly onto the surface of the monolithic BC sample, which retained its original porous structure after carbonization. The wetting process was recorded in real time, and the contact angle was monitored until the droplet was completely absorbed. Each measurement was repeated three times at different locations on the sample surface to ensure reproducibility. For the other characteristics, the BC samples were ground into a fine powder using an agate mortar and pestle. The XRD patterns of the samples were determined by the X-ray diffractometer (XRD, D/max-2500, Rigaku Corporation, Tokyo, Japan). The Raman spectrum of the sample was measured by the Raman spectrometer (Labram HR800, HORIBA Jobin Yvon, Palaiseau, France). The infrared spectra of the samples were obtained by a Fourier-Transform Infrared Spectrometer (IR, Nicolet IS10, Thermo Fisher Scientific, Waltham, MA, USA). The X-ray photoelectron spectrometer (AXIS Supra, Kratos Analytical Ltd., Manchester, UK) quantitatively analyzes the elements and their contents on the material surface. The physical adsorption instrument (ASAP2020, BET method, Micromeritics Instrument Corporation, Norcross, GA, USA) was used to measure the specific surface area and pore size distribution of the sample; approximately 100 mg of sample was degassed under vacuum at 150 °C for 12 h prior to nitrogen adsorption–desorption measurements at 77 K. The ultraviolet–vision–near-infrared spectrophotometer (UV/vis/NIR, V-770, JASCO Corporation, Tokyo, Japan) measures the reflection and transmission spectra of samples within the solar energy wavelength range. The infrared camera (FOTRIC225, Shanghai, China) takes thermal imaging photos and obtains temperature change curves. The inductively coupled plasma atomic emission spectrometer (ICP-AES, Atomscan Advantage, Thermo Jarrell Ash Corporation, Franklin, MA, USA) measures the ion concentration before and after seawater desalination.

### 2.4. Water Evaporation Performance Test

The water evaporation experiment adopts a self-built device in the laboratory. The solar simulator equipped with an AM 1.5 G filter was positioned vertically above the evaporator at a fixed distance of 20 cm to provide a calibrated illumination intensity of 1 kW m^−2^ (1 sun), as measured by an optical power meter (CEL-NP2000, Beijing China Education Au-Light Co., Ltd. (CEAULIGHT), Beijing, China). The evaporator consisted of a polytetrafluoroethylene container (inner diameter: 24 mm, height: 15 mm) filled with 20 mL of water or saline solution. The BC sample (thickness: ~5 mm) was placed on the water surface. The mass change due to evaporation was monitored in real time using a high-precision electronic analytical balance (accuracy: 0.0001 g) connected to a computer for continuous data recording at 1 min intervals. Each reported evaporation rate represents the average of at least three independent measurements under identical conditions, with error bars indicating standard deviation. All evaporation experiments were conducted under controlled laboratory conditions, with the ambient temperature monitored and maintained at 24.8 ± 0.6 °C and relative humidity at 50 ± 10% throughout the measurement period. Temperature and humidity were recorded at the beginning and end of each experiment to ensure they remained within these ranges. For clean water collection experiments, a custom-made transparent condensation chamber was placed over the evaporator, and the condensed water was collected and weighed. The ability of photothermal materials to convert absorbed solar energy into thermal energy and thereby cause water evaporation is usually evaluated by the rate of water evaporation and the efficiency of solar energy conversion. The water evaporation rate ($v_{e}$) and the solar energy conversion efficiency (η) are calculated by the following formula [18,19,20]:

(1) $$\nu_{e}=\frac{dm}{S\times dt}$$

(2) $$\eta =\frac{\nu_{e}\times H_{LV}}{P\times C_{opt}}$$

In the formula, the water evaporation rates are the net values, calculated by subtracting the dark evaporation rate measured under identical conditions. m represents the mass of evaporated water, S is the area of the simulated sunlight vertically shining on the evaporation surface (i.e., the area of the container mouth is 4.52 cm^2^), and t is the evaporation time. P is the solar intensity (1 kW m^−2^), Copt is the optical concentration (1 sun), and H_LV_ is the total liquid–vapor phase change enthalpy. According to the literature, for pure water, the heat value we use is 2440 kJ kg^−1^ (at 25 °C) [21]. For cycling stability tests, the same BC sample was used for 20 consecutive evaporation cycles. After each 1-h evaporation cycle under 1 sun illumination, the sample was allowed to cool to room temperature naturally, and the water in the container was replenished to the initial level before starting the next cycle. The evaporation rate was calculated for each cycle to assess any performance degradation.

### 2.5. The Salt Rejection Experiments

To further evaluate the anti-salt deposition performance of the BC, the BC was placed on the surfaces of simulated seawater (3.5 wt% NaCl solution) and high-concentration brine (15 wt% NaCl solution) in polytetrafluoroethylene containers, respectively, and exposed to simulated sunlight with a light intensity of one sun for 12 h. The mass change of water during the evaporation process was monitored by using an electronic analytical balance, and photos of the evaporation system were taken before and after light exposure to evaluate the accumulation of salt.

### 2.6. Solar-Powered Seawater Desalination Performance Test

In order to test the solar water desalination performance of the BC, a laboratory-made solar water desalination device was used to collect the purified water after solar desalination and conduct water quality tests on it. This device has been reported in a previous article [22]. Four concentrations of NaCl aqueous solutions were used to simulate different seawater samples: the Baltic Sea (0.8 wt% NaCl solution), the World Ocean (3.5 wt% NaCl solution), the Red Sea (4.1 wt% NaCl solution), and the Dead Sea (10 wt% NaCl solution). Real seawater samples from the Bohai Sea were collected in Dalian, China for seawater desalination. The concentrations of Na^+^ ions in simulated seawater before and after seawater desalination and the concentrations of different ions (Na^+^, Mg^2+^, K^+^, and Ca^2+^) in real seawater were analyzed by inductively coupled plasma optical emission spectrometer.

To monitor the actual collection volume of water vapor by the device, simulated seawater (3.5 wt% NaCl solution) was added to the evaporator, and the surface of the simulated seawater was covered with the BC. After continuous evaporation for 6 h under the irradiation of sunlight, the condensate water at the bottom of the beaker was collected.

## 3. Results and Discussion

### 3.1. Characterization of the BC

**Figure 1:**
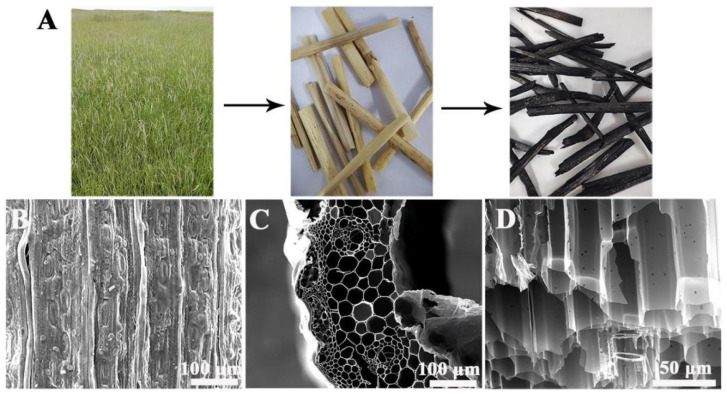
Photographs of natural Elymus nutans (left), ENS after drying (middle), and BC after carbonization (right) (**A**). SEM image of BC epidermis showing surface morphology (**B**). SEM image of lateral vascular bundles and parenchyma cells (**C**). Longitudinal SEM image of parenchyma cells revealing vertically aligned microchannels (**D**).

Figure 1A shows photos of natural Elymus nutans (left), ENS after harvesting and drying (middle), and BC (right) obtained by directly burning Elymus nutans. The stems were obviously carbonized and turned black in color, which is conducive to absorbing sunlight. Scanning electron microscopy (SEM) confirmed that the microstructure of the stem was not damaged after carbonization. As depicted in Figure 1B–D, the SEM images exhibit a well-interconnected, hierarchical porous network comprising macroporous channels and micro-sized pores/textures. The former ensures buoyancy and the latter facilitates a rapid water uptake. This hierarchical porosity is essential for maintaining a continuous water supply to the evaporation interface and minimizing conductive heat loss to the bulk water [23,24].

**Figure 2:**
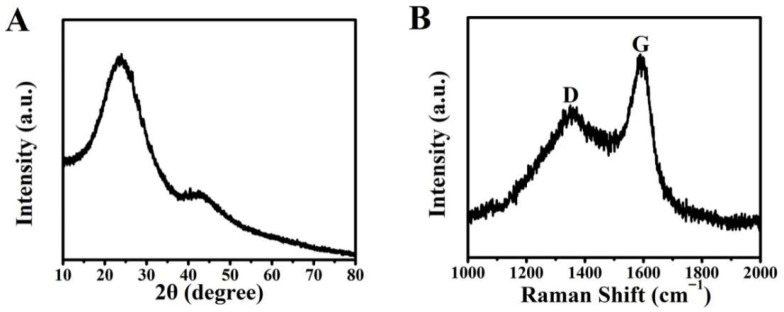
XRD pattern (**A**) and Raman spectrum (**B**) of the BC.

The X-ray diffraction (XRD) pattern of the biomass-derived porous carbon (Figure 2A) exhibits two broad diffraction peaks. The predominant peak centered at approximately 24° is attributed to the (002) plane of graphitic carbon, indicating the formation of stacked graphene-like layers. The weaker and broader peak around 43° corresponds to the (100) plane, reflecting the in-plane structure of the sp^2^ carbon domains. The significant broadening of both peaks confirms the predominantly amorphous nature of the carbon material with limited long-range order, which is characteristic of turbostratic carbon derived from biomass pyrolysis. Nevertheless, the presence of these graphitic crystallites is crucial for efficient photothermal conversion. The Raman spectrum (Figure 2B) displays two prominent peaks: the D band at around 1350 cm^−1^, associated with structural defects and disordered carbon, and the G band at approximately 1590 cm^−1^, originating from the in-plane vibration of sp^2^-bonded carbon atoms in graphitic lattices. The intensity ratio I(_D_)/I(_G_) was calculated to be 0.81. This value indicates a material with a moderate degree of graphitization, comprising a balance of ordered graphitic domains and structural defects. The graphitic domains are crucial for efficient photothermal conversion due to effective electron–phonon coupling, while the defects may contribute to enhanced light absorption through scattering effects.

The coexistence of graphitic and amorphous carbon phases, as evidenced by XRD and Raman analysis, suggests that the material possesses a balanced structure. The graphitic domains facilitate efficient electron–phonon coupling and the rapid thermalization of photoexcited electrons, which is crucial for effective heat generation upon solar irradiation [25]. Concurrently, the amorphous regions and structural defects enhance broadband light absorption through scattering and trap-assisted absorption, a phenomenon also observed in other carbon-based photothermal systems [26]. This synergy is critical for achieving a high photothermal conversion efficiency and sustained thermal localization at the water–air interface during solar desalination.

**Figure 3:**
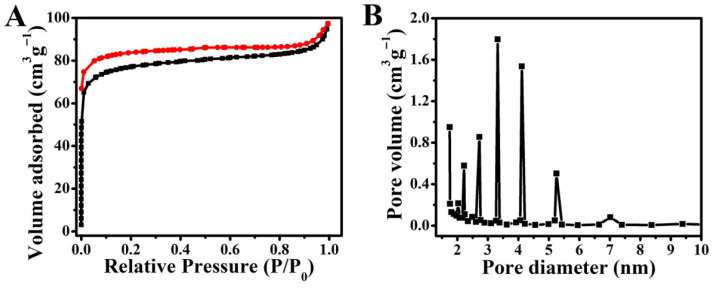
Nitrogen adsorption (black) and desorption (red) isotherms (**A**) and pore size distribution (**B**) of the BC.

The pore structure of biomass-derived porous carbon is crucial for its solar absorption efficiency, water transport kinetics, and salt rejection capacity. The pore architecture of the biomass-derived porous carbon was quantitatively analyzed by N_2_ adsorption–desorption. The isotherm (Figure 3A) is classified as type IV(a) with an H3-type hysteresis loop, confirming a mesoporous structure. The sharp uptake at low P/P_0_ indicates the presence of micropores, which contribute to a specific surface area of 243.1 m^2^ g^−1^. The pore size distribution (Figure 3B) reveals a multimodal hierarchy, featuring prominent mesopores at 3.3 nm and 4.2 nm, a significant volume of micropores (<2 nm), and a total pore volume of 0.68 cm^3^ g^−1^. This hierarchy originates from the inherent biomass microchannels and pores formed during carbonization.

This hierarchical pore configuration is pivotal for high-performance solar desalination, where each level of porosity serves a distinct yet synergistic function. The micropores enhance light harvesting through efficient photon trapping and multiple internal reflections [27]. Concurrently, the mesopores (centered at 3.3 and 4.2 nm) balance capillary-driven water pumping and vapor escape, ensuring a continuous brine supply to the evaporation front. Moreover, the prevalence of mesopores with an average diameter of 4.5 nm provides ample space to accommodate ion clusters and facilitate their back-diffusion, thereby suppressing salt crystallization within the pores. Furthermore, the macroporous framework ensures low-resistance pathways for vapor escape.

Beyond liquid transport, the macroporous framework, as visible in the SEM images, establishes low-resistance pathways for vapor escape and contributes to the material’s self-floating ability. This multi-scale architecture—from sub-nanometer selective channels to micro-sized vapor conduits—creates a synergistic effect that is crucial for the observed high evaporation rate and stable salt rejection [13].

**Figure 4:**
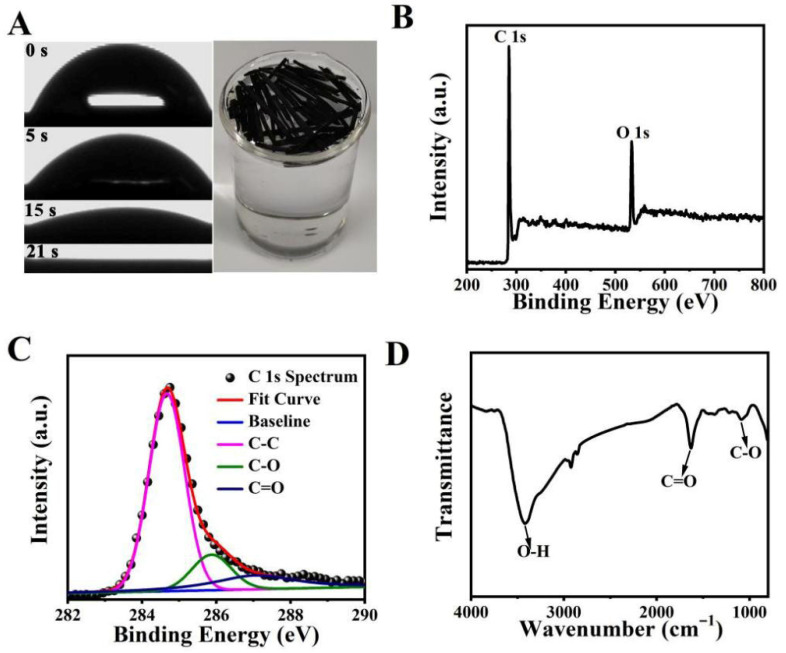
Dynamic contact angle and floating image of the BC (**A**); dynamic contact angle measurement performed on a monolithic BC section, showing complete droplet absorption within 21 s. Inset photograph demonstrates the material’s self-floating capability on water despite its hydrophilic nature. XPS spectra of the BC: full spectrum (**B**) and C1s spectrum (**C**). Infrared spectroscopy of the BC (**D**).

The hydrophilicity of photothermal materials plays a pivotal role in SDVG by facilitating rapid water transport and preventing salt accumulation. The dynamic contact angle measurement (Figure 4A) was performed directly on a section of the as-prepared monolithic BC, retaining its natural porous structure. The droplet was completely absorbed within 21 s, demonstrating the rapid wetting of the hydrophilic carbon surface and capillary wicking through the intrinsic pore network. This seemingly paradoxical combination of complete wettability and self-floating capability arises from the material’s low bulk density, which results from its preserved macroporous architecture. The interconnected macropores trap air, providing buoyancy, while the hydrophilic pore walls facilitate continuous water transport to the evaporation interface—a synergistic design essential for sustained solar desalination performance. Similar phenomena have been reported in other hierarchically structured materials, such as the fully superhydrophilic yet self-floating solar steam generator inspired by seaweed [28]. The surface chemical composition and functional groups of the BC were analyzed by X-ray photoelectron spectroscopy (XPS) and Fourier-transform infrared spectroscopy (FTIR) to further illustrate the hydrophilicity. In Figure 4B, the C1s peak and the O1s peak are discovered. The high-resolution spectra of the C1s peaks (Figure 4C) show three peaks, namely, C-C (284.8 eV), C-O (286.1 eV), and C=O (287.3 eV). Meanwhile, in the FTIR spectrum, as shown in Figure 4D, the three characteristic peaks near 3426 cm^−1^, 1634 cm^−1^, and 1089 cm^−1^ are, respectively, attributed to the O-H stretching vibration, C=O stretching vibration, and C-O stretching vibration. This is consistent with the XPS results. The existence of these functional groups proves that there are a large number of hydrophilic groups in the material. In addition to the hydrophilicity, the BC also features a low density and light weight. These features allow it to float on the surface of water without any other supporting materials, which helps to avoid heating the whole water, promoting the utilization of sunlight and the evaporation of steam.

### 3.2. The Light Absorption and Photothermal Properties of the BC

**Figure 5:**
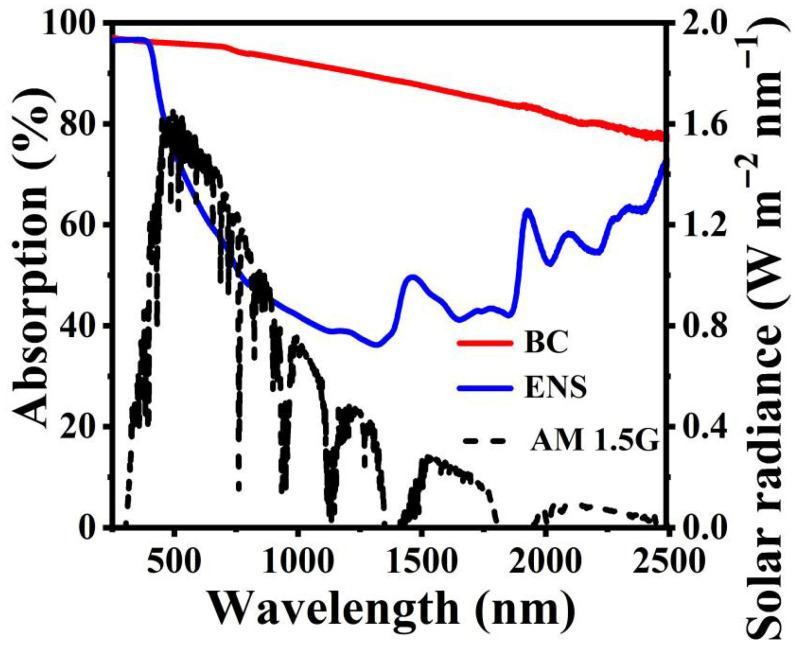
Absorption spectra of the BC, the ENS, and AM 1.5 G.

Strong wide-spectrum absorption is a prerequisite for achieving efficient solar-powered seawater desalination. The transmittance and reflectance of the BC throughout the solar energy spectrum were measured by a UV–vision–near-infrared spectrophotometer. The light absorption rate of the material is calculated through transmittance and reflectance (A = 1 – T − R, where A is the absorption rate, T is the transmittance, and R is the reflectance). As shown in Figure 5, by comparing with the energy distribution of the standard solar spectrum (AM 1.5 G), it can be known that a large proportion of light in the ultraviolet (300–400 nm) and visible light (400–700 nm) regions is absorbed, with light absorption rates of 96.4% and 95.7%, respectively. It was found that 86.2% of the light in the near-infrared (700–2500 nm) region was absorbed. In the solar energy spectrum, the ultraviolet region accounts for approximately 3% of the total energy, the visible light region about 45%, and the near-infrared region about 52%. Calculated proportionally, the solar energy absorption rate of the BC is approximately 90.8%. The light absorption rates of the ENS in the ultraviolet, visible, and near-infrared regions are 96.5%, 71.0%, and 48.8%, respectively. Calculated proportionally, the solar energy absorption rate of the ENS is only about 60.2%. The solar energy absorption rate of the BC is 30.6% higher than that of the ENS. The results prove that the prepared the BC exhibits strong broadband solar absorption, meeting the requirements of high-efficiency SDVG.

**Figure 6:**
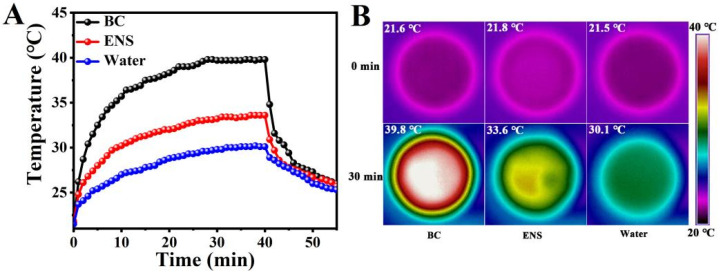
The surface temperature curves (**A**) of pure water, the ENS floating on the water surface, and the BC over time under 1 sun illumination. Infrared thermal imaging images (**B**) of pure water, and the ENS and the BC surfaces after 30 min of irradiation.

In order to verify the photothermal conversion performance of the BC, the temperature variation curve of the material floating on the water surface over time under 1 sun irradiation was recorded by using an infrared thermal imager. As shown in Figure 6A, the surface temperatures surface temperature of the BC rises more rapidly than that of the pure water and the ENS. In 3 min, it rose from 21.6 °C to 30.4 °C for the BC, while pure water and the ENS only reached 24.6 °C and 26.8 °C, respectively. After 30 min of light exposure, the temperature basically reached a balanced state as 30.1 °C for pure water, 33.6 °C for the ENS, and 39.8 °C for the BC, respectively (Figure 6B). The results show that the BC has excellent photothermal conversion performance.

### 3.3. The Water Evaporation Performance of the BC

**Figure 7:**
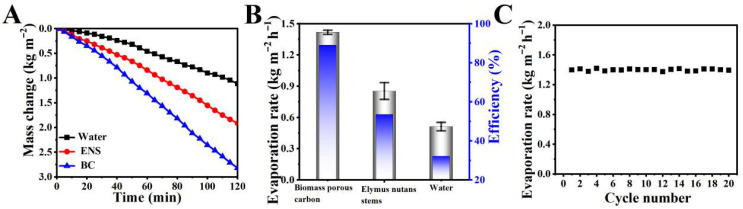
Curves of evaporation of pure water, the ENS, and the BC with exposure time under 1 sun illumination (**A**). The corresponding water evaporation rate and solar energy conversion efficiency (**B**). Evaporation cycle performance of the BC under sunlight (**C**).

The variation in water evaporation with the irradiation time under one sunlight was monitored in real time to evaluate the water evaporation performance. A water evaporation testing device was assembled as shown in Figure 1. Figure 7A shows that the water evaporation increases linearly with the irradiation time. According to Formula (1), the evaporation rate was calculated based on the mass change curve. The evaporation rate of pure water was only 0.51 kg m^−2^ h^−1^. When the ENS were put on the surface, the rate increased to 0.85 kg m^−2^ h^−1^. It significantly increased to 1.41 kg m^−2^ h^−1^ as the BC was used, which is approximately 2.7 times and 1.6 times than that of pure water and the ENS, respectively. According to Formula (2), the solar energy conversion efficiency of pure water, the ENS, and the BC was 32.1%, 53.5%, and 88.9%, respectively (Figure 7B). The efficient water evaporation mainly resulted from the efficient solar energy absorption and utilization, three-dimensional interconnected porous microstructure, and superhydrophilicity. To verify the cycling stability of the BC, as shown in Figure 7C, after cycling 20 times using pure water under one sunlight exposure, the water evaporation rate of the BC can be maintained at about 1.40 kg m^−2^ h^−1^, without a significant decrease, demonstrating the material’s mechanical robustness and reusability under these conditions.

Compared to previously reported biomass-derived evaporators such as carbonized wood [13], mushroom [15], sugarcane [14], and bamboo [17], our material offers a distinct advantage in its combination of one-step fabrication simplicity and robust salt resistance in high-concentration brine. While many reported materials achieve comparable evaporation rates, they often require multi-step processing, chemical modification, or structural reassembly to attain a stable performance in saline conditions. In contrast, the BC derived from Elymus nutans directly leverages its natural hierarchical architecture to maintain stable evaporation in 15 wt% NaCl for over 12 h without any post-treatment, highlighting its potential for practical and scalable applications.

### 3.4. The Salt Deposition Resistance Performance of the BC

**Figure 8:**
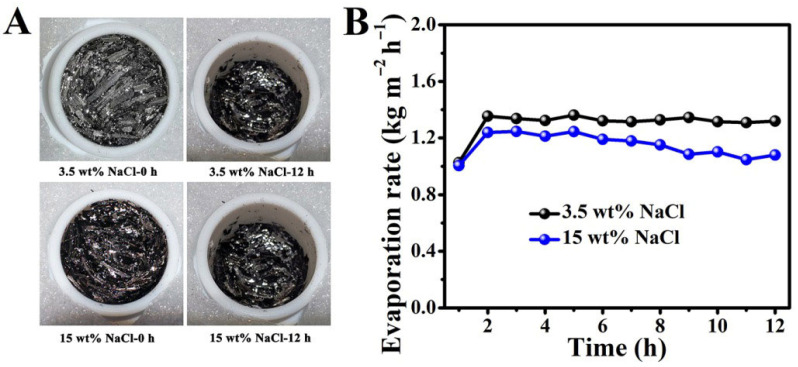
Photograph of the salt precipitation of the BC after 12 h of exposure to the simulated sunlight (one sun) in 3.5 wt% NaCl simulated seawater and 15 wt% NaCl high-salt water (**A**). The variation in evaporation efficiency of the BC during the 12 h evaporation process (**B**).

Salt deposition is the bottleneck problem for achieving continuous and efficient interfacial solar seawater desalination. To evaluate the salt deposition resistance ability of the BC, it was spread over the surfaces of simulated seawater (3.5 wt% NaCl solution) and high-salinity water (15 wt% NaCl solution), and continuously exposed to the simulated sunlight (1 sun). As shown in Figure 8A, after 12 h, no salt crystals appeared on the evaporation surface of the BC in both types of brine. This is because the convective diffusion transport of salt ions and the evaporation rate of water jointly determine the salt concentration on the evaporation surface. During the continuous evaporation process, brine containing salt ions is transported to the evaporation interface through convection to compensate for the evaporated water, resulting in an increase in the ion concentration on the evaporation interface. When the salt ion concentration reaches a saturated state, salt crystals will precipitate. As the BC was spread over the surface, the superhydrophilicity and three-dimensional interconnected multi-level pore structure can provide excellent water transport channels for evaporation. Through capillary force, sufficient water will be pumped to the evaporation interface, thus avoiding the crystallization and precipitation of salt after the salt solution becomes saturated.

The mass change of water during the evaporation process was monitored in real time to evaluate the continuous and efficient evaporation performance of the BC. As shown in Figure 8B, in the 3.5 wt% NaCl simulated seawater, the evaporation rate of the BC remained at about 1.33 kg m^−2^ h^−1^, and the corresponding solar energy conversion efficiency was 83.5%. In 15 wt% NaCl high-salt water, the evaporation rate of the BC remained at approximately 1.16 kg m^−2^ h^−1^, and the corresponding solar energy conversion efficiency was 72.9%. The reason for the decrease in evaporation efficiency in the high-concentration brine is that the evaporation enthalpy of high-concentration brine is higher. In the above two kinds of brine, the water evaporation rate and solar energy conversion efficiency of the BC did not decrease significantly within 12 h.

The results show that the prepared BC can not only inhibit salt deposition, but also maintain a relatively stable and efficient evaporation performance in simulated seawater and even in high-concentration brine. Longer-term stability tests and the post-testing characterization of salt effects on material properties will be essential for future practical validation. These aspects represent important directions for our subsequent research.

### 3.5. The Solar-Powered Seawater Desalination Performance of the BC

**Figure 9:**
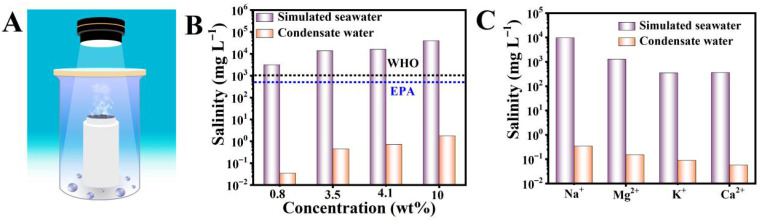
Schematic of a lab-made evaporating and condensing device for solar desalination (**A**). Concentration of Na^+^ in four simulated seawater samples and the corresponding condensate water collected by the interfacial solar seawater desalination system based on the BC (**B**). The concentrations of four primary ions (Na^+^, Mg^2+^, K^+^, and Ca^2+^) before and after actual seawater desalination (**C**).

To evaluate the performance of the BC solar-powered seawater desalination, the concentrations of various ions in the condensate water were tested. Firstly, to collect the condensate water, a laboratory-made solar-powered seawater desalination device for the evaporation and condensation of water was designed. As shown in Figure 9A, this device consists of three parts: a solar simulator, an evaporator, and a condenser. The evaporator is composed of a polytetrafluoroethylene container filled with seawater and the BC covering the surface of the seawater. The evaporator is placed in a sealed condenser (the top of the condenser is made of quartz glass). In the evaporation container, the BC floats on the surface of simulated seawater, such as the Baltic Sea (0.8 wt% NaCl solution), the World Ocean (3.5 wt% NaCl solution), the Red Sea (4.1 wt% NaCl solution), and the Dead Sea (10 wt% NaC solution), and the real Bohai seawater. Under the sunlight, the steam is produced by the evaporation of water through the interface, and then it condensed into water drops accumulating in the condensation chamber. Then, the water was collected to test the concentration of Na^+^, Mg^2+^, K^+^, and Ca^2+^. As shown in Figure 9B, the Na^+^ concentration in the condensate water from the four types of simulated seawater was much lower than the drinking water standards stipulated by the World Health Organization (WHO) and the United States Environmental Protection Agency (EPA). And the concentration of Na^+^, Mg^2+^, K^+^, and Ca^2+^ in the condensate water from the real seawater was likewise lower than the standards and approximately three orders of magnitude lower than the original seawater, which can be seen in Figure 9C. The concentrations of the detected major ions were reduced to levels significantly below the WHO guideline values for drinking water.

By collecting and weighing the condensate water after the simulated seawater was continuously exposed to sunlight for 6 h, it was calculated that the actual water production of the BC solar seawater desalination device was 5.78 L m^−2^. This means that, if each square meter of the BC works continuously for 6 h, the water production can meet the drinking water needs of two normal adults for one day. The results show that the BC has potential application value in the field of interfacial solar seawater desalination.

## 4. Conclusions

In this work, a self-floating biomass porous carbon with robust salt resistance was successfully prepared via the direct, one-step carbonization of Elymus nutans stems. The material’s low density, wide-spectrum absorption, natural three-dimensional porous microstructure, and excellent wettability synergistically enable high-performance solar-driven interfacial desalination. Under 1 sun illumination, the evaporator achieves an evaporation rate of 1.41 kg m^−2^ h^−1^ with an 88.9% solar-to-vapor conversion efficiency, approximately 2.7 times higher than that of pure water. Crucially, it maintains a stable performance in 3.5 wt% and 15 wt% NaCl solutions for over 12 h without salt clogging, and successfully desalinates real seawater to meet drinking water standards.

The prepared biomass porous carbon represents a sustainable, low-cost, and abundant photothermal material that can be obtained through simple carbonization, laying a foundation for large-scale application, particularly in remote and underdeveloped regions. Future work will focus on extending salt resistance to higher concentrations, the systematic optimization of material parameters, outdoor testing under real solar conditions, and a comprehensive life-cycle assessment to further advance this material platform toward practical implementation in solar desalination and related applications such as wastewater treatment and steam sterilization.

## Data Availability

The original contributions presented in this study are included in the article. Further inquiries can be directed to the corresponding author.
